# Caveolin-3: therapeutic target for diabetic myocardial ischemia/reperfusion injury

**DOI:** 10.1186/s10020-025-01117-5

**Published:** 2025-02-26

**Authors:** Xinyu Wen, Yanwei Ji, Hepeng Tang, Zhenshuai Jin, Wating Su, Lu Zhou, Zhong-Yuan Xia, Lin Li, Shaoqing Lei

**Affiliations:** 1 Department of Anesthesiology, Renmin Hospital of Wuhan University, Wuhan City, China; 2 Department of Anesthesiology, Affiliated RenHe Hospital of China, Second Clinical Medical College, Three Gorges University, Yichang, Hubei Province China

**Keywords:** Caveolin-3, Myocardial ischemia/Reperfusion injury, Diabetes, Cardioprotection

## Abstract

Myocardial ischemia/reperfusion (I/R) injury is a major global health problem with high rates of mortality and disability, which is more severe in patients with diabetes. Substantial researches have documented that diabetic myocardium are more susceptible to I/R injury, but many current intervention strategies against myocardial I/R injury have limited effectiveness in diabetic hearts. Caveolin-3 (Cav-3) is the signature protein of caveolae and serves as a signal integration and transduction platform in the plasma membrane of cardiomyocytes, which plays a vital role in myocardial functions, metabolism and protection of multiple conditioning strategies against I/R injury. Nevertheless, numerous studies have revealed that the expression of Cav-3 is impaired in diabetic hearts, which contributes to increased vulnerability of myocardium to I/R injury and resistance to protective conditioning strategies. In this review, we outline the basic structure and function of Cav-3, emphatically present the unique role of Cav-3 as a signal integration and transduction element in diabetic myocardial I/R injury and discuss its therapeutic perspective in strategies against myocardial I/R injury in diabetes.

## Introduction

Diabetes mellitus is a chronic, metabolic diseases. Its global prevalence was estimated to be more than 536.6 million patients in 2021, and it was expected to reach 1.31 billion by 2050 (Anonymous 2023; Sun et al. 2022a). Diabetes mellitus can adversely affect multiple organs and systems, leading to systemic complications, of which ischemic heart disease is the major cause of death and disability in patients (Einarson et al. 2018; Hausenloy et al. 2017). Timely restoration of blood flow is the most effective strategy for ischemic heart disease, especially for acute myocardial infarction, whereas reperfusion may elicit additional myocardial damage and acute heart failure, termed as ischemia/reperfusion (I/R) injury (Hausenloy et al. 2017; Lejay et al. 2016). Unfortunately, diabetic hearts have an increased susceptibility to I/R injury and are insensitive to multiple cardioprotective conditioning (e.g., ischemic preconditioning) (Lejay et al. 2016; Penna et al. 2020), whereas the exact mechanisms remain to be elucidated.

Caveolin-3 (Cav-3), mainly distributed in myocardial and skeletal muscle cells, is a subtype of caveolae’ iconic protein caveolins (Parton 2018). Cav-3 have essential functions in cardiovascular system, involving cellular structure maintenance, signal transduction, metabolism and ion channels regulation and vesicular transport (Schilling et al. 2015; Parton 2018). As a signal integration platform, Cav-3 is considered to be a key regulator of multiple endogenous cardioprotective signaling (e.g., the reperfusion injury salvage kinase (RISK) pathway and survivor activating factor enhancement (SAFE) pathway) against myocardial I/R injury (Yang et al. 2016). Thus, improving Cav-3 expression, which can mimick the protective effects of ischemic preconditioning, is found to alleviate I/R-induced myocardial injury, whereas the injury is aggravated after Cav-3 deletion (Tsutsumi et al. 2008; Wang et al. 2012). These findings suggest Cav-3 is essential for maintaining myocardial tolerance against I/R injury. In addition, abnormal changes in Cav-3 are considered to be closely related to the occurrence and progression of diabetes, involving the regulation of insulin signaling, glucose and lipid metabolism, redox reaction and mitochondrial homeostasis (Capozza et al. 2005; Murfitt et al. 2015), etc. Moreover, we and others have shown impaired expression of Cav-3 in diabetic hearts (Lei et al. 2013; Penumathsa et al. 2008), which may be a key reason for increased susceptibility to I/R injury in diabetic hearts. Therefore, strategies targeting Cav-3 may have promising treatment effects. This review aims to clarify the role of Cav-3 in diabetic myocardial I/R injury and explore its potential as a therapeutic target.

## Caveolae and Cav-3

Caveolae, originally described by Palade in 1953 and Yamada in 1955 (Eichi Yamada 1955), are 50- to 80-nm bulb-shaped uncoated invaginations of the plasma membrane. They are a striking morphological features of mammalian cells with variable densities in different tissues. Incredibly high density of caveolae have been found in muscles, adipocytes, and endothelia, while in some tissues, such as liver and renal proximal tubules, they may be rare or absent (Parton 2018). Caveolae appear as single pits or caveola rosettes, a multilobed structure composed of several caveolae, by transmission electron microscopy (Miguel A. Del Pozo 2021). The morphological transformation of caveolae is in response to the change of membrane tension, thereby protecting cells from mechanical stress damage (Lo et al. 2015). In addition, the characteristic lipid composition and proteins make caveolae highly dynamic and participate in various cellular processes, including vesicular transport (e.g., endocytosis and transcytosis), lipid homeostasis, mechanoprotection and signal transduction (Park et al., 2021b; Yang et al. 2016). Mutations in caveolae proteins or other pathological changes of caveolae have been implicated in various diseases including cardiac hypertrophy, muscular dystrophy, abnormal glucose metabolism, lipodystrophy, pulmonary dysfunction, prostate and breast cancer (Parton 2018; Yin et al. 2016), etc.

**Fig. 1:**
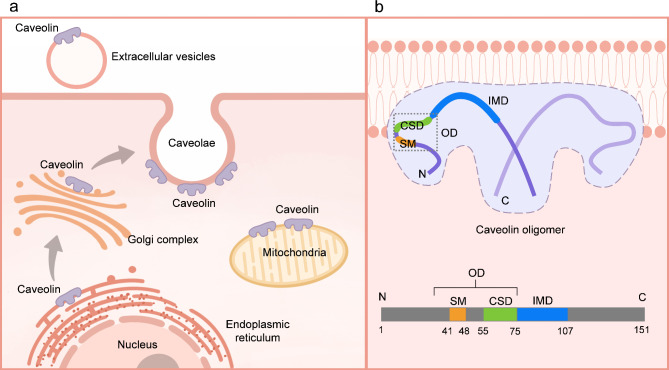
The cellular components’ distribution and structure of Cav-3. **a**. This diagram summarizes the cellular distribution of Cav-3. Cav-3 is transferred to plasma membrane after synthesis and processing in endoplasmic reticulum and Golgi, and it can also transfer to mitochondria and extracellular vesicles. **b**. This diagram shows the structure of caveolin oligomer and the main domains of Cav-3. Cav-3 has 151 amino acid residues and four main domains: IMD, CSD, OD, and SM. The OD, containing the CSD and SM, is represented with the dashed box. Caveolins can insert into plasma membrane via the IMD with their N- and C-terminal facing the cytoplasm. They are linked to each other through their OD domains to form a disc-shaped oligomeric complex on the membrane, and their C-terminals can be spirally assembled into barrel-like channels. The CSD is mainly responsible for the interaction of Cav-3 with other signaling molecules. IMD: intramembrane domain, CSD: caveolin scaffolding domain, OD: oligomerization domain, SM: signature motif

Caveolins are signature proteins of caveolae and play essential roles in caveolae formation. They are detectable in different subcellular organelles including plasma membrane, Golgi, endoplasmic reticulum, mitochondria and extracellular vesicles (Fig. 1a) (Shah et al. 2020; Siwaponanan et al. 2022). Mammals possess three subtypes of caveolins: Cav-1, Cav-2, and Cav-3. Cav-1 and Cav-2 are widely co-expressed in multiple tissues including adipocytes, endothelial cells, macrophages, and type I pneumocytes, whereas Cav-3 is muscle-specific, primarily expressed in myocardial and skeletal muscle cells. Cav-3 is 65% identical and 85% similar to Cav-1, and they can form homo- and hetero-oligomers, which drive membrane curvature and formation of caveolae on the plasma membrane (Parton 2021). Thus, the loss of Cav-1 or Cav-3 leads to the loss of caveolae in different tissues (Parton 2018).

Cav-3 is identified as a 17 kDa membrane sculpting and lipid-binding protein consisting of 151 amino acid residues. It contains several distinctive domains, including intramembrane domain (IMD), caveolin scaffolding domain (CSD), oligomerization domain (OD) and signature motif (SM) ** (**Fig. 1b). The IMD enables insertion of caveolins oligomers into membrane, inducing membrane curvature and promoting mature caveolae formation via cooperating with other related proteins (Porta et al. 2022). The CSD is essential for membrane association, cholesterol binding, oligomerization and regulation of signal transduction, which dominates the interaction between Cav-3 and a variety of cardioprotective and metabolic signaling molecules, including phosphatidylinositol 3-kinase (PI3K), protein kinase B (Akt), endothelial nitric oxide (NO) synthase (eNOS), protein kinase C (PKC), Mitsugumin 53 (MG53, also named TRIM72), adiponectin receptor 1 (AdipoR1) and G proteins (Lei et al. 2019; Xia et al. 2022; Yang et al. 2021; Zhang et al. 2011), etc. Apart from these classic characterized regions, more and more domains in caveolins have been described in recent studies, including pin motif, spoke region and β strand. Most of them are currently thought to contribute to the formation of the disc-shaped oligomeric complexes of caveolins on the plasma membrane that is essential for caveolins function (Park et al., 2021b; Parton and Collins 2022; Porta et al. 2022). Future work of caveolins structural change, functional sites and post-translational modification will deepen our understanding of caveolins and relative pathophysiological processes.

## Role of Cav-3 in diabetes

Cav-3 is closely involved in insulin signaling, glucose metabolism and lipid homeostasis, dysfunction of which are common manifestation of diabetes. It has been shown that mice with genetic ablation of Cav-3 exhibit impaired insulin signaling, abnormal glucose and lipid metabolism, adiposity, and insulin resistance with ligand-induced insulin receptor instability in skeletal muscle, which has similar features to type 2 diabetes (Capozza et al. 2005; Oshikawa et al. 2004). Although the density of the insulin receptor remains unchanged in skeletal muscle of Cav-3 ablated mice, the expression of insulin receptor and activation of downstream signaling molecules are reduced after insulin stimulation, and re-expression of Cav-3 via gene transduction can rescue this abnormality (Capozza et al. 2005; Oshikawa et al. 2004). Moreover, overexpression of Cav-3 in liver improves insulin sensitivity, insulin receptor signaling and glucose metabolism with increased glycogen synthesis in KKAy mice with type 2 diabetes (Otsu et al. 2010). These findings suggest that Cav-3 is a stabilizer and enhancer of insulin sensitivity and insulin signaling. In addition, insulin also affects the expression of Cav-3. In in vitro experiments, long-term insulin exposure independently leads to up-regulation of Cav-3 expression (Russell et al. 2020), further implicating a reciprocal regulation between Cav-3 and insulin signaling.

Cav-3 is also involved in glucose uptake and thus affects the pathological course of diabetes. In in vitro and in vivo studies, Cav-3 can enhance glucose transporter 4 (GLUT-4) translocation into the plasma membrane through insulin-dependent or independent signaling, thereby promoting glucose uptake (Penumathsa et al. 2008; Shang et al. 2017). GLUT-4 vesicle trafficking is crucial for glucose uptake in cardiomyocyte, skeletal muscle and adipose cells, which is mediated by complex mechanisms including insulin stimulation, exercise, stress, anoxia, ischemia and catecholamines. It has been shown that GLUT-4 targets caveolae and co-localizes with Cav-3 in the membrane, and Cav-3 can facilitate GLUT-4 translocation via differential modulation of the adenosine monophosphate-activated protein kinase (AMPK)/Akt/eNOS signaling pathway (Murfitt et al. 2015; Penumathsa et al. 2008). The impaired Akt-mediated translocation of GLUT-4 contributes greatly to insulin resistant, which can be alleviated by resveratrol that increase Cav-3 expression (Tan et al. 2012). Thus, the depressed Cav-3 expression accompanied by reduction of GLUT-4 is likely to be the key pathological mechanism of diabetes (Penumathsa et al. 2008).

## Role of Cav-3 in myocardial I/R injury

As a highly aerobic and energy demanding tissue, myocardium is extremely sensitive to I/R injury. This pathological process is closely related to calcium overload, oxidative stress, endoplasmic reticulum (ER) stress, activation of inflammatory cascade, mitochondrial dysfunction, apoptosis and pyroptosis (Ferdinandy et al. 2023; Kalogeris et al. 2016), etc. As a characteristic transmembrane protein of myocardium, Cav-3 has been demonstrated to play an essential role in protection against myocardial I/R injury. The expression and distribution of Cav-3 change in response to myocardial I/R injury (Ratajczak et al. 2003). Mice with myocyte-specific Cav-3 overexpression exhibit substantially enhanced tolerance to myocardial I/R injury, while Cav-3 ablation exhibit a higher sensitivity to myocardial I/R injury, manifested by larger infarct size, higher levels of cardiac troponin I (cTnI), swollen mitochondria, distended myofibrils (Tsutsumi et al. 2008; Wang et al. 2012). Similarly, age-dependent reductions of Cav-3 distribution at the plasma membrane and caveolae abundance may contribute to decreased ischemic tolerance with age (Ratajczak et al. 2003). These studies indicate that Cav-3 is critical for maintain myocardial tolerance to I/R injury.

The current strategies with considerable protection against myocardial I/R injury in preclinical models could be divided into ischemic conditioning, pharmacological conditioning and physical therapy (Schilling et al. 2015; Wu et al. 2021). According to the timing of intervention, they can be mainly classified as preconditioning (intervening before ischemia), perconditioning (intervening during ischemia) and postconditioning (intervening before or in the early minutes of reperfusion). Ischemic conditioning, including ischemic preconditioning, ischemic postconditioning and remote ischemic conditioning, is considered as a typical and effective treatment against I/R injury in animal experiments, and has entered clinical translation (Bell et al. 2016; Hausenloy et al. 2017; Kan et al. 2023). The clinical transformation also maintains great challenge: differences in the observation indicators and timing, sample selection preferences, disease type and extent, surgical procedures, anesthetic management, drugs application, etc. Though two important large scale clinical trials about remote ischemic conditioning did not demonstrate a positive outcome (Hausenloy et al. 2015; Meybohm et al. 2015), remote ischemic conditioning has advantages in protective mechanism, safety, convenience, and intervention timing, which suggests its great potential and research value in protection against myocardial I/R injury. The short-term and non-lethal ischemic conditioning activates complex endogenous protective mechanisms that make tissues better resistant to I/R injury, including the RISK pathway (e.g., PI3K/Akt and extracellular signal-regulated kinases 1 and 2 (ERK1/2) cascades), SAFE pathway (e.g., Janus-activated kinase (JAK)/signal transducer and activator of transcription 3 (STAT3) cascades), eNOS, and p38 mitogen-activated protein kinase (p38 MAPK) (Heusch 2015; Schilling et al. 2015; Zhang et al. 2011), etc.

Interestingly, it has been shown that myocardial protection induced by conditioning strategies is weakened or eliminated in Cav-3 ablated mice, while cardiac-specific overexpression of Cav-3 can mimic the protection of ischemic preconditioning against I/R injury with increased basic phosphorylation of Akt and glycogen synthase kinase-3β (GSK3β) (Schilling et al. 2015; Tsutsumi et al. 2008). Additionally, Cav-3-MG53-PI3K complex-mediated activation of RISK pathway and Cav-3-mediated transport of ERK1/2 to the mitochondria have been proposed to be important mechanisms in the protection conferred by ischemic postconditioning, which can suppress GSK3β-mediated opening of mitochondrial permeability transition pore (MPTP) and subsequent apoptosis and necrosis (Hernández-Reséndiz and Zazueta 2014; Zhang et al. 2011). It can be seen that Cav-3 plays a crucial role in ischemic conditioning-mediated endogenous protection.

An improved understanding of the mechanisms underlying I/R injury and endogenous protection has resulted in extensive researches on pharmacological conditioning, which target the activation of similar pro-survival pathways or directly intervene the pathophysiological process of I/R injury such as oxidative stress, inflammation, autophagy and apoptosis (Ferdinandy et al. 2023; Wu et al. 2021). As a multi-signal regulatory platform involved in myocardial protection, the role of Cav-3 in pharmacological conditioning has received increasing attention. Analyses of the phenotypes of Cav-3 ablated and wild type mice or rats have provided strong evidence for it. The protective effects against myocardial I/R injury of adiponectin, exendin-4, opioids, and isoflurane conditioning are shown to be abolished in Cav-3 ablated models (Tsutsumi et al. 2010a, b, 2014a; Wang et al. 2012). These results suggest that they work in a Cav-3-dependent manner. Furthermore, increased expression of Cav-3 in myocardium is observed after the treatment of melatonin, isoflurane, or geranylgeranylacetone in animal models of ischemic heart failure or I/R (Şehirli et al. 2013; Tsutsumi et al. 2010a, b; Yang et al. 2016), which indicates that Cav-3 is an important effector molecule for pharmacological conditioning. Additionally, Cav-3 has been indicated to have a regulatory role with ATP-sensitive K+ (K_ATP_) channels, the opening of which is an important target for a class of pharmacological preconditioning (e.g., nicorandil) against myocardial I/R injury (Maslov et al. 2023). Sarcolemmal K_ATP_ channels are enriched in caveolae and directly interacts with Cav-3 (Garg et al. 2009), while Cav-3 is found to negatively regulate K_ATP_ channels endocytic recycling (Huo et al. 2023). It reveals a new regulatory way of Cav-3, but how they work and coordinate during I/R and in protection strategies requires further exploration. In conclusion, as an important signal integration platform on the plasma membrane, Cav-3 has great potential in cardioprotection against I/R injury, and targeting the expression or activation of Cav-3 is a promising therapeutic strategy.

## Role of Cav-3 in diabetic myocardial I/R injury

Cav-3 integrates and regulates a wide variety of signals to maintain cellular homeostasis, playing an important protective role in both myocardial I/R injury and diabetes as described above. Substantial experimental and clinical evidence indicates that diabetic hearts are more susceptible to I/R injury and less responsive to established cardioprotective conditioning (Malfitano et al. 2014; Penna et al. 2020; Russell et al. 2017), which may due to the impaired Cav-3 expression in diabetic hearts. Though not being fully clarified, several underlying mechanisms of Cav-3 in diabetic myocardium have received accumulating attention, including oxidative stress, ER stress, mitochondrial dysfunction, autophagy, inflammation and apoptosis, which will be described separately below.

### Cav-3 and oxidative stress

Excessive oxidative stress is the key mechanism of I/R injury, and is also considered to be a feature of diabetes (Moldogazieva et al. 2019; Peng et al. 2022). During myocardial I/R, reactive oxygen species (ROS) production increases during ischemia and exacerbates when the blood supply is restored, inducing intense oxidative damage, metabolic disorders, inflammation, impaired cellular function and apoptosis (Kalogeris et al. 2016). In the context of diabetes, elevated basal oxidative stress detrimentally affects myocardial resistance to I/R injury. There may be several reasons for the enhancement of basal oxidative stress in diabetic hearts. First, the increased free fatty acids in diabetes activates cardiac peroxisome proliferator-activated receptor α (PPARα), which further promotes the uptake and metabolism of free fatty acids at the transcription level, resulting in abnormal energy metabolism, lipotoxicity and enhanced oxidative stress (Chong et al. 2017). Second, the accumulation of advanced glycation end products (AGEs) in diabetes activates signaling cascades via interaction with the receptors for AGEs (RAGE), thereby exacerbating oxidative stress and inflammation (Bansal et al. 2023; Moldogazieva et al. 2019). Third, the impaired insulin signaling aggravates metabolic disorders and energy imbalance, indirectly promoting oxidative stress and weakening myocardial resistance to I/R injury (Clerk and Sugden 2022). Fourth, the prolonged oxidative stress-induced reduction in eNOS activation and NO production further exacerbates redox imbalance and myocardial injury (Kalogeris et al. 2016). Additionally, the antioxidant defense is also impaired in long-term diabetes. Chronic hyperglycemia during diabetes downregulates myocardial nuclear factor erythroid 2-related factor 2 (Nrf2), a key regulator of cellular antioxidant response, resulting in decreased expression of another important cytoprotective enzyme, heme oxygenase-1, and ultimately lead to excessive oxidative stress (Gao S 2016).

**Fig. 2:**
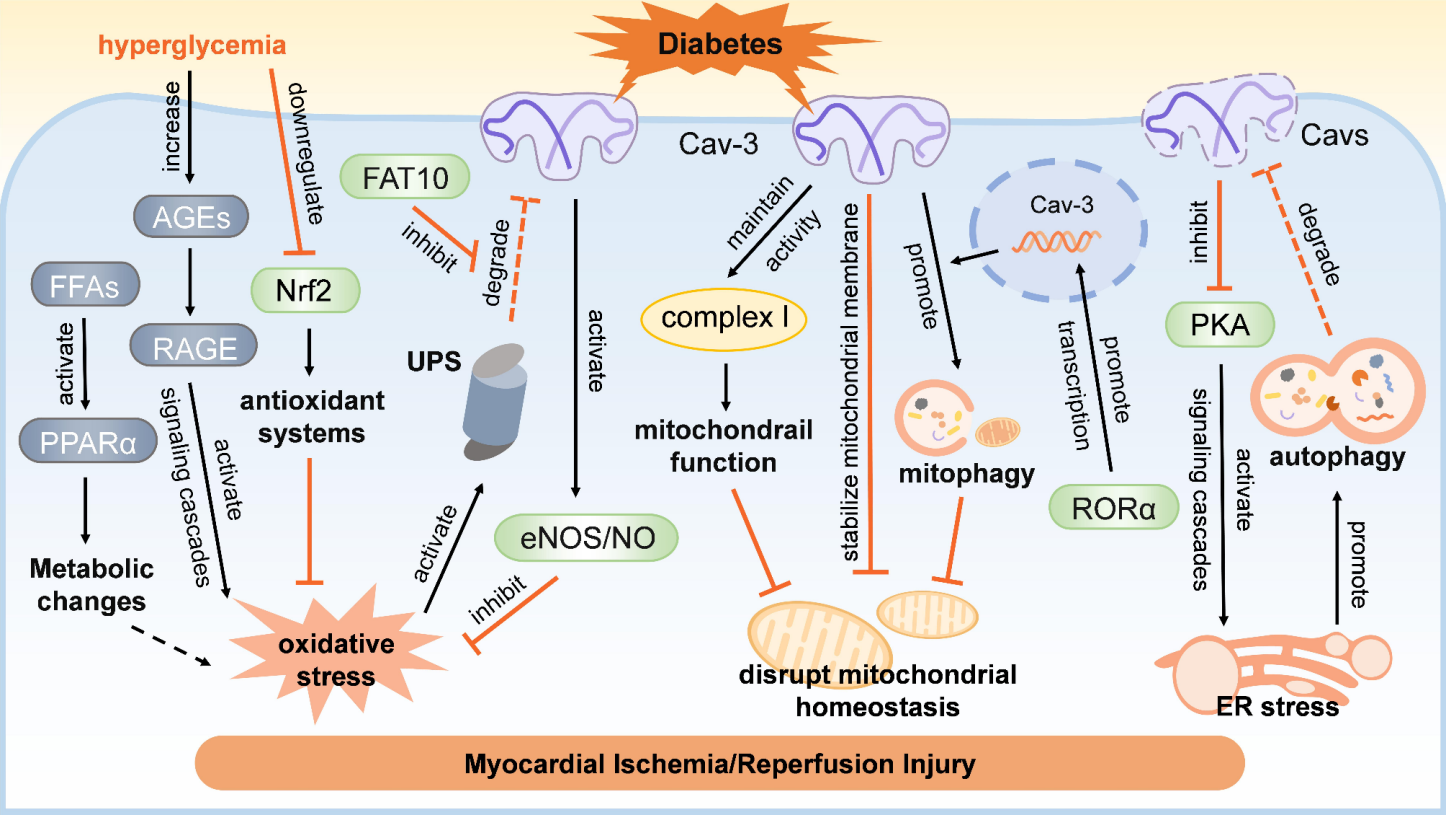
Regulation of Cav-3 on oxidative stress, ER stress, mitochondrial homeostasis in diabetic myocardial I/R injury. This figure demonstrates that Cav-3 regulates oxidative stress, ER stress, mitochondrial homeostasis through a variety of signals to exert protective effects against myocardial I/R injury. Diabetes impairs myocardial Cav-3 expression, resulting in increased vulnerability to I/R injury. Cav-3: caveolin-3, ER: endoplasmic reticulum, FFAs: free fatty acids, PPARα: peroxisome proliferator-activated receptor α, AGEs: advanced glycation end products, RAGE: receptors for AGEs, Nrf2: nuclear factor erythroid 2-related factor 2, FAT10: F adjacent transcript 10, UPS: the ubiquitin-proteasome system, NO: nitric oxide, eNOS: endothelial NO synthase, PKA: protein kinase A, RORα: retinoic acid-related orphan nuclear receptor

The impaired Cav-3 expression might be closely related to excessive oxidative stress and increased vulnerability to I/R injury in diabetic myocardium (Fig. 2) (Xia et al. 2022; Zhao et al. 2017). Our previous study has shown that increased oxidative stress is accompanied with reduced Cav-3 expression in diabetic hearts, and treatment with antioxidant N-acetylcysteine increases Cav-3 levels while restoring cardioprotective eNOS/NO signaling to normal levels (Su et al. 2016). This suggests that reduction of Cav-3 in diabetic hearts is likely owe to increased oxidative stress, and a certain degree of recovery of Cav-3 may be achieved through antioxidant treatment. Additionally, our further study has found that knockdown of Cav-3 by siRNA increases the levels of superoxide anion (O_2_^•−^) in cardiomyocytes, a main oxidizing substance during I/R, and improving Cav-3 expression attenuates ROS production and postischemic injury in diabetes (Lei et al. 2019; Su et al. 2016). This raises the possibility of a bidirectional relationship between Cav-3 and oxidative stress. The specific mechanism responsible for inhibition of Cav-3 by oxidative stress remains to be elucidated, but it’s thought to be related to activation of the ubiquitin-proteasome system (UPS). The UPS is considered to be the main machinery responsible for degrading cytosolic proteins in the repair system and is found to be overactive during oxidative stress (Qiu et al. 2022a). It has been shown that Cav-3 mutants can be degraded by UPS rather than lysosomes (Galbiati et al. 2000). F adjacent transcript 10 (FAT10), a member of the ubiquitin-like-modifier family, is shown to exert a protective effect against I/R injury by stabilizing Cav-3 and antagonizing its ubiquitination-mediated degradation in cardiomyocytes (Zhou et al. 2018). These findings suggest that UPS is an important degradation pathway for Cav-3. However, both oxidative stress and ubiquitination are complex processes, and the specific mechanism by which their interaction affecting Cav-3 needs to be further investigated.

### Cav-3 and ER stress

ER stress is a cellular response to the accumulation of misfolded and unfolded proteins and disorders of calcium homeostasis, which initiates the unfolded protein response (UPR), ER-associated degradation and autophagy to maintain cellular homeostasis and protein quality control (Ren et al. 2021). When ER homeostasis is challenged, the adaptive UPR will be activated to restore protein homeostasis via reducing transcription and translation, promoting protein folding and removing abnormal proteins, which is mediated by three crucial enzymes: protein kinase RNA-like ER kinase (PERK), activating transcription factor 6 (ATF6) and inositol-requiring enzyme 1α (IRE1α) (Hetz et al. 2020). When ER chaperone binding immunoglobulin protein (BiP/GRP78), a sensor of ER stress, disassociates away from these three proteins, the rescue program mediated by these three branches will be initiated. However, under sustained and strong stimulation by I/R, excessive ER stress can trigger maladaptive response resulting in apoptosis, which is mainly mediated by IRE1α and PERK proapoptotic pathways (Kalogeris et al. 2016; Souza-Neto et al. 2022; Zhu and Zhou 2021). In diabetes, metabolic disturbances induce persistent ER stress, and ultimately leading to cardiac remodeling, myocardial dysfunction and increased vulnerability to I/R injury (Lemmer et al. 2021; Yang et al. 2015). Consequently, mitigating excessive ER stress represents an effective strategy to enhance the tolerance of diabetic hearts to I/R injury (Li et al. 2019; Shi et al. 2015).

Growing evidence suggests a cross-talk of caveolins and ER stress (Fig. 2). It is shown that Cav-1 scaffold domain peptide can be internalized into the cytoplasm and accumulated in the ER, thereby alleviating ER stress by restoring the basal level of IRE1α (Komatsu et al. 2022). Additionally, Cav-1 can localize to ER-mitochondrial interface and interferes their contact by inhibiting protein kinase A (PKA) during the early phase of ER stress, thus affecting mitochondrial response to ER stress (Bravo-Sagua et al. 2019). Recent study shows that deficiency of Cav-1 promotes cholesterol accumulation and exacerbates ER stress in a murine model of metabolic dysfunction-associated steatotic liver disease, while supplementing CSD by intraperitoneally injection, a stable analog of the active domain of Cav-1, can alleviate ER stress and it-mediated pyroptosis (Xu et al. 2025). Given the high homology between Cav-3 and Cav-1, it is reasonable to believe they exert similar effects. This inference is further supported by research demonstrating a notable elevation in ER stress in P104L mutant Cav-3 mice (Kuga et al. 2011), indicating a potential negative regulatory role of Cav-3 in ER stress. Sevoflurane preconditioning is shown to alleviate acute myocardial I/R-induced ER stress in a Cav-3-dependent way (Zhang et al., 2022b). Considering the impaired Cav-3 expression in diabetic hearts and its crucial involvement in myocardial resistance to I/R injury, we speculate that deficiency of Cav-3 may exacerbate ER stress, thereby leading to increased susceptibility of diabetic hearts to I/R injury. The underlying mechanism needs further investigation. On the other hand, ER stress can mediate the degradation of caveolins by promoting autophagy. Cav-1 expression was significantly decreased accompanied by overactive ER stress-mediated autophagy in particulate matter-induced pulmonary fibrosis model, while inhibition of ER stress could attenuate both autophagy and the reduction of Cav-1 (Liu et al. 2023). Although there is currently no direct evidence supporting the relationship between the reduction of Cav-3 in diabetic hearts and that mechanism, it may be verified in future studies.

### Cav-3 and mitochondrial homeostasis

Mitochondria are the most important organelles in maintaining myocardial function, with their damage being a central event in myocardial I/R injury. During I/R, oxidative burst and Ca^2+^ overload trigger the opening of MPTP, mitochondrial swelling and disintegration, which lead to the release of mitochondrial DNA, ROS and cytochromes c, ultimately resulting in collapse of mitochondrial network and myocardial injury (Jiang et al. 2021; Mendoza et al. 2024). It’s worth noting that growing researches pay attention to the role of imbalance of mitochondrial quality control in myocardial I/R injury (Bai et al. 2023). Mitochondrial quality control, mainly including mitochondrial fusion, fission, biogenesis and mitophagy, is an adaptive response to cope with external stimuli and maintain mitochondrial homeostasis via regulating mitochondrial quality and quantity (Wang and Zhou 2020). During myocardial I/R, severe damage in mitochondria exceeds the threshold of mitochondrial quality control capabilities, leading to disruption of mitochondrial homeostasis and energy metabolism, which ultimately results in mitochondrial fragmentation, apoptosis and necroptosis (Wang and Zhou 2020). Thus, interventions aimed at maintaining mitochondrial homeostasis have great potential in alleviating myocardial I/R injury, including inhibiting mitochondrial fission, promoting fusion and FUN14 domain containing 1 (FUNDC1)-mediated mitophagy, and conducting mitochondrial transplantation (Bai et al. 2023; Maneechote et al. 2017; Park et al. 2021a; Vásquez-Trincado et al. 2016; Wang and Zhou 2020; Yang et al. 2019; Zhang et al. 2019). However, in the context of diabetes, hyperglycemia and insulin resistance perturb myocardial energy metabolism and mitochondrial homeostasis, leading to structural abnormalities of mitochondrial, increased mitochondrial fission, and decreased fusion and mitophagy (Ding et al. 2017; Rovira-Llopis et al. 2017; Zheng et al. 2021). Further studies demonstrate that inhibiting mitochondrial fission by selectively downregulating dynamin-related protein 1 in diabetic hearts can not only attenuate I/R injury but also restore the protective effect of sevoflurane postconditioning (Ding et al. 2017; Yu et al. 2021). These evidences suggest that perturbations of mitochondrial homeostasis may weaken the ability of mitochondrial quality control to withstand stress, thus leading to increased susceptibility of diabetic hearts to I/R injury, and restoring mitochondrial homeostasis is a promising strategy against diabetic myocardial I/R injury.

Interestingly, growing evidence have shown that Cav-3 is a crucial regulator of mitochondrial homeostasis. A striking metabolic and morphological shift of mitochondria is shown in Cav-3 ablated myoblasts, including deficit of mitophagy and excessive mitochondrial fragmentation due to imbalance of fission and fusion (Shah et al. 2020). Notably, Cav-3 has been detected in mitochondria, indicating a more direct link between Cav-3 and mitochondria (Fridolfsson et al. 2012). The translocation of Cav-3 to mitochondria enhances mitochondrial respiratory function, stabilizes mitochondrial membrane and increases their tolerance to cellular stress (Fridolfsson et al. 2012; Wang et al. 2014). Therefore, promoting the transfer of Cav-3 or Cav-3-associated molecules to mitochondria is a crucial mechanism for certain cardioprotective conditioning against myocardial I/R injury, including isoflurane, δ-opioid and ischemic postconditioning (Fridolfsson et al. 2012; Hernández-Reséndiz and Zazueta 2014; Wang et al. 2014, 2020). Additionally, the retinoic acid-related orphan nuclear receptor α (RORα), a potential cardioprotective target for I/R injury, has been shown to promote cardiomyocyte mitophagy by directly enhancing Cav-3 transcription (Beak et al. 2021). These studies suggest that Cav-3-mediated maintenance of mitochondrial homeostasis is an important strategy against myocardial I/R injury. However, Cav-3 is significantly downregulated in diabetic hearts, whereas its cardiac-specific upregulation is shown to ameliorate diabetes-induced mitochondrial dysfunction via restoring the activity of mitochondrial complex I (Guo et al. 2024). Overall, Cav-3 is vital for maintaining mitochondrial homeostasis, and its reduction contributes to decreased tolerance to I/R injury and insensitivity to protective conditioning in diabetic hearts via disrupting mitochondrial homeostasis (Fig. 2).

### Cav-3 and autophagy

Autophagy is an essential adaptation and defense mechanism to maintain cellular physiological function and homeostasis via removing and recycling damaged or excessive cellular components or eliminating invading pathogens (Mizushima and Komatsu 2011; Vargas et al. 2023). As for terminally differentiated cardiomyocyte, autophagy is particularly important for cellular survival and homeostasis in adverse conditions, such as diabetes, I/R and aging (Kalogeris et al. 2016; Kim and Lee 2014; Sciarretta et al. 2018). In diabetic hearts, multiple factors including metabolic disorders, insulin resistance, oxidative stress and inflammation, attribute to impaired autophagy, which is considered to be an important cause of cardiovascular complications in diabetes (Dewanjee et al. 2021). The involvement of autophagy in myocardial I/R injury is more complex. During ischemia, autophagy is activated by energy deprivation and multiple stress, which facilitates the clearance of damaged and detrimental components and partly compensates for substrate depletion, limiting cellular damage and death; whereas excessive activation of autophagy during reperfusion may elicit detrimental influence, which may attribute to overwhelming consumption of cellular constituents or autophagic cell death (Daniels et al. 2019; Sciarretta et al. 2018). Our previous studies have shown that cardiac autophagy is downregulated in 8-week diabetic rats, and interventions to restore autophagy significantly alleviate myocardial I/R injury (Qiu et al., 2022b). However, a consensus on cardiac change of autophagy and its role in myocardial I/R injury in diabetes to date have not been achieved, which may attribute to the type of diabetes, disease course, species, ways of modeling, or selectivity in autophagy (Chen et al. 2021; Sun et al., 2022b; Wang et al. 2018; Yu et al. 2018), etc. Thus, maintaining autophagy at an appropriate level may be the effective strategy against myocardial I/R injury in the context of diabetes.

**Fig. 3:**
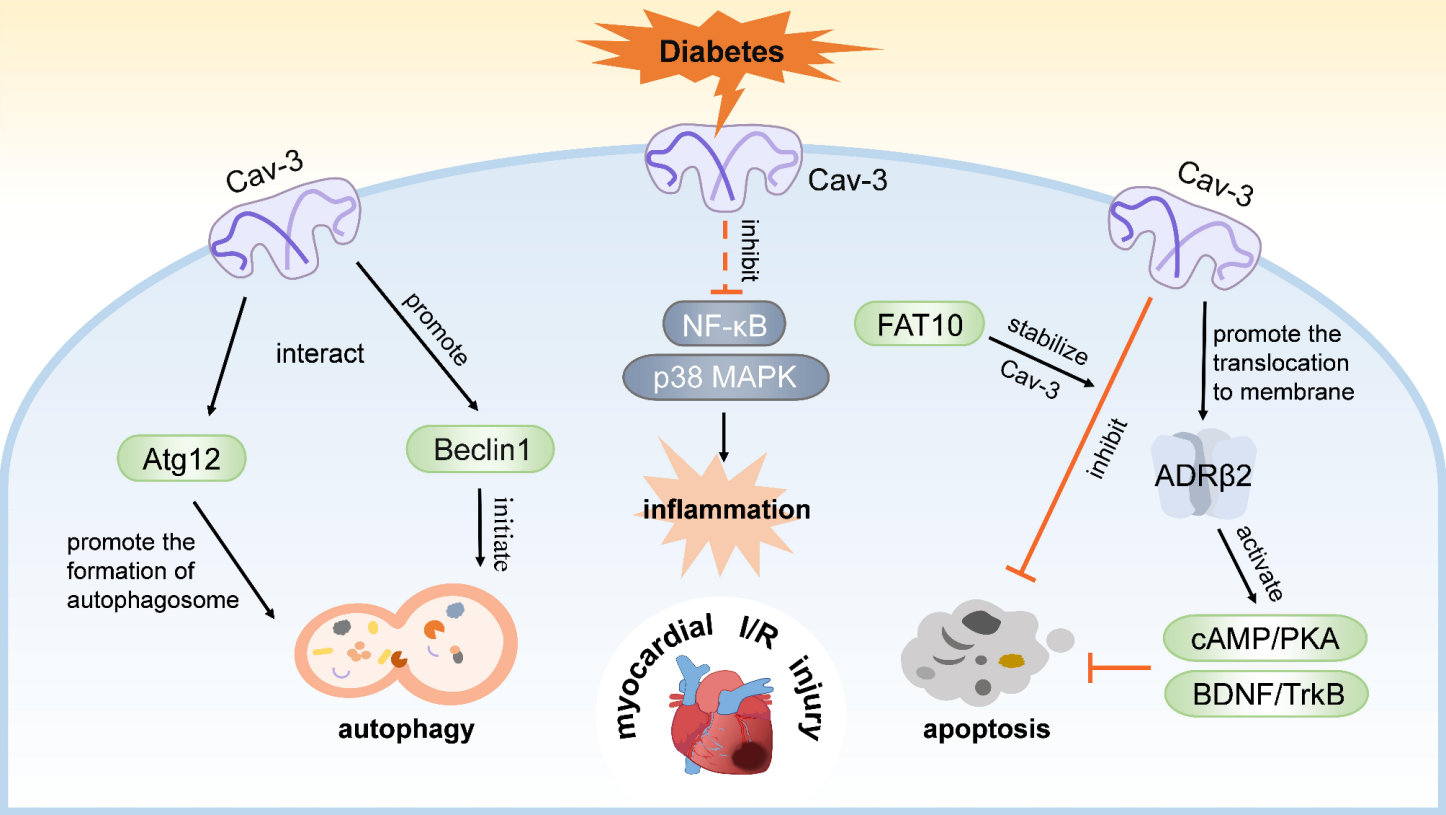
The regulation of autophagy, inflammation and apoptosis in diabetic myocardial I/R injury by Cav-3. This figure shows the regulatory roles of Cav-3 on multiple pathophysiological mechanisms in myocardial I/R injury, including autophagy, inflammation and apoptosis. The impaired expression of Cav-3 in diabetic hearts is a potential cause of more severe I/R injury. Cav-3: caveolin-3, Beclin1: Bcl-2-interacting protein1, Atg12: autophagy-related protein 12, FAT10: F adjacent transcript 10, ADRβ2: adrenergic receptor β2, cAMP: cyclic adenosine monophosphate, PKA: protein kinase A, BDNF: brain-derived neurotrophic factor, TrkB: tropomyosin-related kinase receptor B

Cav-3 is a potential regulator of autophagy. It has been found that Cav-3 respectively co-immunoprecipitates with the classic autophagy-initiating protein Bcl-2-interacting protein1 (Beclin1), and autophagy-related protein 12 (Atg12), a key component of the ubiquitin-like conjugation system, thereby promoting autophagosome formation, and the interaction between Cav-3 and them are reduced in Cav-3-deficient cardiomyocytes (Fig. 3) (Kassan et al. 2016). Moreover, Cav-3 overexpression can enhance autophagy with increased Beclin1, microtubule-associated protein 1 light chain 3-II (LC3-II) and decreased p62 (also named Sequestosome 1) under starvation and rapamycin stimulation, while these effects are limited in Cav-3 knockdown cardiomyocytes (Kassan et al. 2016). Further researches have revealed that the overactive autophagy mediated by upregulated Beclin1 during reperfusion is found to be reduced in Cav-3 knockdown cardiomyocytes after simulated I/R, accompanied by decreased Beclin1 and increased apoptosis (Shi et al. 2019). The linkage between Cav-3 and autophagy-associated proteins suggests a potential regulatory role of Cav-3 in autophagy during myocardial I/R. Interestingly, substantial evidence has shown dysregulation of autophagy and decreased Cav-3 in diabetic hearts (Dewanjee et al. 2021; Lei et al. 2013; Murfitt et al. 2015), while the reduction of Cav-3 may be an important reason for autophagy impairment. Relative insufficiency in autophagy may render diabetic hearts more vulnerable to I/R injury, and interventions targeting Cav-3 to maintain a proper level of autophagy in diabetic myocardial I/R may be an exciting subject for future research.

### Others

In addition to the mechanisms described above, Cav-3 is also involved in other pathological processes in diabetic myocardial I/R injury, such as inflammation and apoptosis (Fig. 3). The activation of inflammatory signaling pathways is a typical process in diabetes and I/R injury (Algoet et al. 2023; Rohm et al. 2022). Although the association between Cav-1 and inflammation has been extensively studied in various diseases (de Almeida 2017; Zhang et al. 2022a), research focusing on the link between Cav-3 and inflammation remains limited. Early studies exploring the roles of Cav-3 in lymphocyte activation have shown that Cav-3 ablation results in reduction of B-cell population-percentage and decreased expression of IL-2 and IL-17 in T-helper cells, which suggests a potential regulation of Cav-3 on B-cell expression, T-cell cytokine production and inflammation (Tran et al. 2015). Further studies demonstrate that the localization of Cav-3 on the plasma membrane is essential for cardioprotection of the TIR/BB-loop mimetic AS-1 (hydrocinnamoyl-L-valylpyrrolidine), which can inhibit myocardial inflammation by disrupting I/R-induced interaction between interleukin-1 receptor and myeloid differentiation factor 88 (Hu et al. 2017; Cao et al. 2009). Additionally, puerarin (daidzein-8-C-glucoside), a traditional Chinese medicine extract, can upregulate Cav-3 expression and inhibit the proinflammatory transcription factor nuclear factor-κB (NF-κB) and p38 MAPK pathways in H9C2 cardiomyocytes treated with high glucose and high-fat, whereas its protective effects are abolished by Cav-3 silencing (Tian et al. 2023). This indicates that Cav-3 may exert anti-inflammatory effects by negatively regulating p38 MAPK-NF-κB pathway. However, these results need further verification in animal models, which differ significantly from cellular models. As a signal platform on the plasma membrane, the interaction of Cav-3 and inflammatory signaling in diabetic myocardial I/R injury needs further investigation.

Apoptosis, a widely studied programmed cell death in I/R injury, has also been reported to be negatively regulated by Cav-3. Under hypoxic stress, knockdown of Cav-3 aggravates cardiomyocyte apoptosis with a significant increase in cleaved caspase-3 (Zhou et al. 2018). Moreover, FAT10 can stabilize Cav-3 expression by antagonizing its ubiquitination-mediated degradation, thereby inhibiting hypoxia-induced cardiomyocyte apoptosis (Peng et al. 2013; Zhou et al. 2018). Similarly, restoring impaired Cav-3/eNOS/NO signaling is found to be a critical step for nitroxyl to alleviate high glucose-induced apoptosis (Sun et al. 2020). Another study demonstrates that overexpression of Cav-3 inhibits high glucose or hypoxia/reoxygenation-induced apoptosis in H9C2 cardiomyocytes (Gong et al. 2020). These results show that Cav-3 has an anti-apoptotic effect. Further investigation of the underlying mechanism reveals that overexpression of Cav-3 promotes the localization of adrenergic receptor β2 (ADRB2) to the membrane, thereby activating the cyclic adenosine monophosphate (cAMP)/PKA and brain-derived neurotrophic factor (BDNF)/tropomyosin-related kinase receptor B (TrkB) signaling pathways to mitigate cell apoptosis and hypoxia/reoxygenation injury in cardiomyocytes under high glucose condition (Gong et al. 2020). The anti-apoptotic effect of n-3 polyunsaturated α-linolenic acid is found to rely on the upregulation of Cav-3, which prevents the internalization of caveolar tumor necrosis factor receptor 1 (Carotenuto et al. 2013). In addition, Cav-3 has been found to play an important role in anti-apoptotic machinery of some preconditioning against I/R, such as propofol, empagliflozin and dapagliflozin (Nikolaou et al. 2022; Zhu et al. 2017), which involves the activation of STAT3 and Akt pathway. However, how Cav-3 regulates and what the specific apoptosis pathway it regulates in diabetic myocardial I/R injury require future investigation.

Taken together, Cav-3, as an important signal integration and regulatory element, is involved in multiple pathological mechanisms of diabetic myocardial I/R injury and shows promising therapeutic potential.

## Role of Cav-3 in conditioning strategies against diabetic myocardial I/R injury

As previously mentioned, conditioning strategies (e.g., ischemic conditioning, pharmacological conditioning) represent promising therapies against myocardial I/R injury and has been the subject of extensive research (Table 1) (Tian et al. 2020; Tsutsumi et al., 2010b; Wang et al. 2012). Unfortunately, the protective effects of various conditioning strategies are diminished or lost in diabetic hearts, including ischemic conditioning, insulin preconditioning, remifentanil preconditioning and isoflurane postconditioning (Baranyai et al. 2015; Kim et al. 2010; Nakadate et al. 2017; Penna et al. 2020; Wang et al. 2016), etc. Though the reasons are not fully understood, these phenomena may be related to the impairment of insulin signal and endogenous protective mechanisms (Penna et al. 2020). Interestingly, numerous studies have suggested that the lost protection of some conditioning strategies in diabetic hearts is likely attributable to reduction of Cav-3, since they work in a Cav-3-dependent manner. Specifically, long-term diabetes impairs the expression of Cav-3, leading to the silencing of multiple endogenous protective pathways, thereby ultimately weakening the protection of ischemic conditioning (Li et al. 2016; Tian et al. 2020). The critical role of Cav-3 in many conditioning strategies has been demonstrated in ablation models, and restoring Cav-3 expression in diabetic hearts may reinstate the protective effects of those conditioning. Our study has shown that remifentanil preconditioning-induced protection is abolished in diabetic hearts due to hyperglycemia-induced oxidative stress and impaired Cav-3 expression, while antioxidant treatment with N-acetylcysteine improves Cav-3 expression and Cav-3-dependent Akt and STAT3 activation, thereby restoring remifentanil preconditioning-induced cardioprotection (Lei et al. 2019). These results suggest great potential of restoring Cav-3 expression in diabetic hearts for salvage the cardioprotection of those strategies. Apart from influence on Cav-3 expression, recent studies have revealed a new sight on its post-translational modification. An important nitration site of Cav-3, Tyr73, is identified by mass spectrometry (Meng et al. 2023). The nitration is facilitated in the prediabetic hearts, and results in dissociation of Cav-3/insulin receptor-β complex and Cav-3/AdipoR1 complex, which contributes to cardiac insulin/adiponectin resistance (Meng et al. 2023). Incidentally, insulin and adiponectin conditioning had been proved to be effective strategies in preclinical studies, which are closely related Cav-3 (Sato et al. 2014; Wang et al. 2012). Further work of this research shows that phenylalanine substitution of Tyr73 can restore it via avoiding nitration at this site (Meng et al. 2023). This reminds us, in addition to expression and distribution, flexible control of post-translational modification will be an interesting direction of future research.

Currently, studies have also found that some pharmacological conditioning strategies less affected by diabetes have potential link with Cav-3. ADRB2 agonist isoproterenol treatment can alleviate high glucose-aggravated hypoxia/reoxygenation injury in H9C2 cardiomyocytes via enhancing the activity of cAMP/PKA and BDNF/TrkB signaling pathways, whereas Cav-3 overexpression has been shown to share the same mechanism via promoting the localization of ADRB2 (Gong et al. 2020). However, these results need to be further validated in animal models. Similarly, dexmedetomidine, a potent α-2 adrenergic receptor agonist, is shown to ameliorate myocardial I/R injury in the context of diabetes via stimulating PI3K/Akt pathway, a typical protective signaling modulated by Cav-3 (Cheng et al. 2018; Sun et al., 2022b). These results imply that Cav-3 plays a regulatory role in these strategies, and their preserved protection in diabetic hearts may attribute to their positive effects on Cav-3 expression. Specifically, propofol has been indicated to attenuates post-hypoxic mitochondrial damage and cell death in cardiomyocytes during high glucose via upregulating Cav-3 and it-mediated Akt and STAT3 activation (Deng et al. 2018). Overall, conditioning strategies modulating Cav-3 expression, distribution or post-translational modification are the promising approach for diabetic myocardial I/R injury.

However, although many conditioning strategies modulating Cav-3 expression are effective in experimental models, there are still many challenges in clinical translation, which may be due to the differences between animal diabetes models and human diabetes, and many conditioning strategies are difficult to simulate in humans. Additionally, there is a lack of effective means to directly interfere with Cav-3 expression in clinical practice, as there are no agonists and inhibitors targeting Cav-3. Thus, developing clinically applicable and effective strategies that can regulate myocardial Cav-3 in a site-directed approach will become an important research topic in the future, such as direct or indirect gene therapy for upregulating Cav-3 expression, treatment with mimicry peptide of Cav-3 critical domains CSD, or targeting specific molecules interacting with Cav-3, etc.

**Table 1:** The role of Cav-3 in conditioning against (diabetic) myocardial I/R injury

Conditioning	Diabetes Models	(S) I/*R* Models	Mechanism	References
IPC		Rats	Increases expression of Cav-3, GLUT-4, p-eNOS, and p-Akt, and enhances interaction between Cav-3 and GLUT-4 in the membrane	(Koneru et al. 2007)
		Rats and MG53 KO mice	Restores MG53 expression, and enhances MG53-dependent interaction of Cav-3 and PI3K and their-mediated activation of RISK pathway	(Cao et al. 2010)
		Cav-3 KO mice	In subsarcolemmal mitochondria, IPC increases eNOS and Cav-3 level, and promotes Cav-3-associated eNOS/NO transport to regulate S-nitrosylation of mitochondrial proteins	(Sun et al. 2015)
IPTC		MG53 KO mice	Increases interaction between Cav-3 and PI3K in a MG53-dependent manner, thereby enhancing RISK pathway activity	(Zhang et al. 2011)
		Dilated cardiomyopathy rats	Promotes Cav-3-mediated translocation of ERK1/2, GSK3β and Akt to mitochondria, thereby inhibiting the opening of MPTP	(Hernández-Reséndiz and Zazueta 2014)
		Rats	Caveolae and Cav-3 mediate the translocation of activated ERK1/2 to subsarcolemmal and interfibrillar mitochondria	(García-Niño et al. 2017)
		Rats	Promotes redistribution of Cav-3 through actin-cytoskeleton network	(Correa et al. 2023)
Isoflurane preconditioning		Cav-3 KO mice and rat cardiac myocytes	Cav-3 and caveolae are essential for isoflurane-induced cardiac protection	(Horikawa et al. 2008)
		Cav-3 KO mice	Cav-3 is essential for delayed anesthetic preconditioning; delayed anesthetic preconditioning increases plasma membrane localization and interaction of Cav-3 and GLUT-4	(Tsutsumi et al. 2010a)
		Cardiac-specific Cav-3 OE mice	Increases localization of Cav-3 to plasma membrane and mitochondria in an inhibitory G protein signaling-dependent manner	(Wang et al. 2014)
Sevoflurane preconditioning		Cav-3 KO mice and cardiac-specific AMPKα2 dominant negative OE mice	Inhibits COX-2 in a Cav-3 dependent and AMPK-independent manner	(Zhao et al. 2013)
Helium postconditioning		Rats	15 min of helium postconditioning increases Cav-3 level in the membrane and activation of ERK1/2 and Akt	(Flick et al. 2016)
Propofol preconditioning		Rats and Cav-3 KD H9C2 cardiomyocytes	Attenuates I/R-induced proteasome degradation of Cav-3	(Zhu et al. 2017)
Opioids preconditioning		Cav-3 KO mice and cardiac-specific Cav-3 OE mice	Cav-3 is essential for opioid-induced preconditioning	(Tsutsumi et al., 2010b)
		Cav-3 KO/OE mice and HL-1 cardiomyocytes	Cav-3 is essential for cardiac protection induced by acute opioid receptor activation, but not sustained ligand-activated preconditioning	(See Hoe et al. 2014)
		Mice	Promotes Cav-3 transfer to plasma membrane and mitochondria via inhibitory G protein signaling, thereby inhibiting the opening of MPTP	(Wang et al. 2020)
		Cardiac-specific Cav-3 KD rats	Increases ERK1/2 phosphorylation and inhibits apoptosis in a Cav3/µ receptor complex-dependent manner	(Guo et al. 2025)
Tocotrienol preconditioning		Rats	γ tocotrienol inhibits interaction of p38MAPKβ with Cav-3	(Das et al. 2008)
Adiponectin postconditioning		Cav-3 KO mice	Cav-3/AdipoR1 interaction is vital for adiponectin-initiated AMPK-dependent or-independent intracellular cardioprotective signaling	(Wang et al. 2012)
Exendin-4 preconditioning		Cav-3 KO mice and rat cardiac myocytes	Interaction of Cav-3 and glucagon-like peptide-1 receptor is essential for exendin-4-induced protection	(Tsutsumi et al. 2014a)
GGA preconditioning		Cav-3 KO mice	Increases caveolae and Cav-3 in membrane	(Tsutsumi et al., 2014b)
AS-1 postconditioning		Mice, rat ventricular myocytes and Cav-3 KD H9C2 cardiomyocytes	Induce cellular redistribution of Cav-3 to plasma membrane	(Hu et al. 2017)
SGLT-2i preconditioning		Mice	Short-term administration of empagliflozin and dapagliflozin increase Cav-3 and fibroblast growth factor-2 level, thereby activating STAT3 and RISK pathway	(Nikolaou et al. 2022)
IPTC	STZ-induced T1DM	Rats, adiponectin KO mice and H9C2 cardiomyocytes	Chronic hyperglycemia abrogates IPTC protection by impairing AdipoR1/Cav-3 signaling leading to an inability to activate mitochondrial STAT3	(Li et al. 2016)
Propofol postconditioning	high glucose	H9C2 cardiomyocytes	Propofol attenuates high glucose and hypoxia/reoxygenation induced reductions in Akt and STAT3 activation in a cav-3-dependet manner	(Deng et al. 2018)
Remifentanil preconditioning	STZ-induced T1DM	Cav-3 KD rats	Hyperglycemia-induced oxidative stress impairs Cav-3 expression and it-modulated PI3K/Akt and JAK2/STAT3 signaling, abrogates remifentanil-mediated protection	(Lei et al. 2019)
Ruboxistaurin preconditioning	STZ-induced T1DM	Rats	Inhibits diabetes-induced increased PKCβ2 to restore Cav-3/Akt signaling	(Liu et al. 2015)
N-acetylcysteine preconditioning	STZ-induced T1DM/high glucose	Rats, rat cardiac myocytes and Cav-3 KD H9C2 cardiomyocytes	N-acetylcysteine restores Cav-3/eNOS signaling in diabetic rats attenuates myocardial dysfunction and I/R injury	(Su et al. 2016)
Isoproterenol postconditioning	High glucose	Cav-3 OE H9C2 cardiomyocytes	Cav-3 OE promotes the localization of ADRB2 to the membrane, thereby enhancing the activity of the cAMP/PKA and BDNF/TrkB signaling pathways	(Gong et al. 2020)
Insulin postconditioning	High-fat diet-induced T2DM	Cav-3 KO mice and Cardiac-specific Cav-3 mutation mice	Diabetic nitrative modification of Cav-3 at Tyr73 impair Cav-3/insulin receptor-β and Cav-3/AdipoR1 associations, thereby resulting in cardiac insulin/adiponectin resistance in the prediabetic heart	(Meng et al. 2023)

*** (S) I/R***: simulated ischemia/reperfusion, ***IPC***: ischemic preconditioning, ***IPTC***: ischemic postconditioning, ***GLUT-4***: glucose transporter 4, ***p-eNOS***: phospho-endothelial nitric oxide synthase, ***p-Akt***: phospho-protein kinase B, ***KO***: knockout, ***OE***: overexpression, ***KD***: knockdown, ***MG53***: mitsugumin 53, ***PI3K***: phosphatidylinositol 3-kinase, ***RISK***: reperfusion injury salvage kinase, ***ERK1/2***: extracellular signal-regulated kinases 1 and 2, ***GSK3β***: glycogen synthase kinase-3β, ***MPTP***: Mitochondrial permeability transition pore, ***COX-2***: cyclooxygenase-2, ***AMPK***: adenosine monophosphate-activated protein kinase, ***p38MAPKβ***: p38 mitogen-activated protein kinase β, ***AdipoR1***: adiponectin receptor 1, ***STAT3***: signal transducer and activator of transcription 3, ***GGA***: geranylgeranylacetone, ***AS-1***: hydrocinnamoyl-L-valylpyrrolidine, ***SGLT-2i***: sodium glucose co-transporter-2 inhibitors, ***STZ***: streptozotocin, ***JAK2***: Janus-activated kinase 2, ***PKCβ2***: Protein kinase C β2, ***T1DM***: type 1 diabetes, ***T2DM***: type 2 diabetes, ***ADRB2***: adrenergic receptor β2, ***cAMP***: cyclic adenosine monophosphate, ***PKA***: protein kinase A, ***BDNF***: brain-derived neurotrophic factor, ***TrkB***: tropomyosin-related kinase receptor B

## Conclusion

Ischemic heart disease is the main cardiovascular complication and the primary cause of disability and death in patients with diabetes. The diabetic myocardium is more sensitive to I/R injury and is resistant to various conditioning, which make it urgent to clarify the pathogenic mechanism of myocardial I/R injury in the setting of diabetes, and to discover effective protection strategies against it. Cav-3, an important myocardial protective protein, is impaired in diabetic hearts, which may be related to increased oxidative stress, ER stress, disturbed mitochondrial homeostasis and autophagy, etc. Cav-3 has been shown to participate in the regulation of multiple endogenous protective pathways, playing a key role in susceptibility to diabetic myocardial I/R injury and restoration the sensitivity of conditioning in diabetic hearts. Treatments targeting restoration of Cav-3 have great research prospects. At this stage, researches on Cav-3 are still in the animal and cell experiments, and it needs to be clinically confirmed in the future. However, the translation from experimental models to clinical practice remains challenging.

## Data Availability

No datasets were generated or analysed during the current study.

## Abbreviations

I/R: Ischemia/reperfusion
Cav: Caveolin
RISK: Reperfusion injury salvage kinase
SAFE: Survivor activating factor enhancement
IMD: Intramembrane domain
CSD: Caveolin scaffolding domain
OD: Oligomerization domain
SM: Signature motif
PI3K: Phosphatidylinositol 3-kinase
Akt: Protein kinase B
NO: Nitric oxide
eNOS: Endothelial nitric oxide synthase
PKC: Protein kinase C
MG53: Mitsugumin 53
AdipoR1: Adiponectin receptor 1
GLUT-4: Glucose transporter 4
AMPK: Adenosine monophosphate-activated protein kinase
ER: Endoplasmic reticulum
cTnI: Cardiac troponin I
ERK1/2: Extracellular signal-regulated kinases 1 and 2
JAK: Janus-activated kinase
STAT3: Signal transducer and activator of transcription 3
p38 MAPK: p38 mitogen-activated protein kinase
GSK3β: Glycogen synthase kinase-3β
MPTP: Mitochondrial permeability transition pore
PPARα: Peroxisome proliferator-activated receptorα
AGEs: Advanced glycation end products
RAGE: Receptors for advanced glycation end products
Nrf2: Nuclear factor erythroid 2-related factor 2
UPS: Ubiquitin-proteasome system
FAT10: F adjacent transcript 10
UPR: Unfolded protein response
PERK: Protein kinase RNA-like endoplasmic reticulum kinase
ATF6: Activating transcription factor 6
IRE1α: Inositol-requiring enzyme 1α
BiP/GRP78: Binding immunoglobulin protein
PKA: Protein kinase A
FUNDC1: FUN14 domain containing 1
RORα: Retinoic acid-related orphan nuclear receptorα
Beclin1: Bcl-2-interacting protein1
Atg12: Autophagy-related protein 12
LC3-II: Light chain 3-II
NF-κB: Nuclear factor-κB
cAMP: Cyclic adenosine monophosphate
BDNF: Brain-derived neurotrophic factor
TrkB: Tropomyosin-related kinase receptor B
COX-2: Cyclooxygenase-2
GGA: Geranylgeranylacetone
AS-1: Hydrocinnamoyl-L-valylpyrrolidine
SGLT-2i: Sodium glucose co-transporter-2 inhibitors
STZ: Streptozotocin
T1DM: Type 1 diabetes
T2DM: Type 2 diabetes
ADRB2: Adrenergic receptor β2
K_ATP_: ATP-sensitive K+
